# Identification and characterization of human testis derived circular RNAs and their existence in seminal plasma

**DOI:** 10.1038/srep39080

**Published:** 2016-12-13

**Authors:** Wei-Wei Dong, Hui-Min Li, Xing-Rong Qing, Dong-Hui Huang, Hong-Gang Li

**Affiliations:** 1 Family Planning Research Institute/Center of Reproductive Medicine, Tongji Medical College, Huazhong University of Science and Technology, Wuhan, 430030, P. R. China; 2 Wuhan Tongji Reproductive Medicine Hospital, Wuhan, 430030, P. R. China

## Abstract

Circular RNAs (circRNAs) have emerged as novel molecules of interest in gene regulation as other noncoding RNAs. Though they have been explored in some species and tissues, the expression and functions of circRNAs in human reproductive systems remain unknown. Here we revealed the expression profiles of circRNAs in human testis tissue using high-throughput sequencing. The conformation of these testis-derived circRNAs in seminal plasma was also investigated, aiming to provide a non-invasive liquid biopsy surrogate for testicular biopsy. We predicted >15,000 circRNAs in human testis, with most of them (10,792; 67%) new. In all the 5,928 circRNA forming genes, 1,017 are first reported by us to generate circRNAs. Interestingly, these genes are mostly related to spermatogenesis, sperm motility, fertilization, etc. The sequence feature, chromosome location, alternative splicing and other characteristics of the circRNAs in human testis were also explored. Moreover, we found that these testis-derived circRNAs could be stably detected in seminal plasma. Most of them were probably bound with proteins in seminal plasma and were very stable at room temperature. Our work has laid the foundations to decipher regulation mechanisms of circRNAs in spermatogenesis and to develop circRNAs as novel noninvasive biomarkers for male infertile diseases.

Spermatogenesis is a complex process which includes the following: differentiation of spermatogonia stem cell into spermatogonia cell, spermatocyte, round spermatid and elongated sperm^1^. During spermatogenesis, sequential and coordinated expression of stage-specific genes is essential for the normal development of male gametes^2^. Gene regulation at transcriptional, post-transcriptional and epigenetic levels are observed in this process^3^,^4^. Noncoding RNAs such as microRNAs (miRNAs), PIWI-interacting RNAs (piRNAs) and long non-coding RNAs (lncRNAs) have long been found as important post-transcriptional regulators of gene expression in multiple stages of spermatogenesis^5^. miRNAs regulate spermatogenesis by affecting the stability or in some cases the translation of specific mRNAs^6^. piRNAs are efficient repressors of transposon element expression in spermatogenesis^7^. lncRNAs can act as recruiters for transcription factors to inhibit or activate stage-specific expression of spermatogenetic genes^8^. Abnormalities in the timely and adequate expression of these noncoding RNAs will result in abnormities in spermatogenesis such as hypospermatogenesis or mature arrest^7^,^8^,^9^.

Recently, a new class of noncoding RNAs, the circular RNAs (circRNAs) has aroused researchers’ attention. CircRNAs are a class of RNAs that, unlike linear RNAs, form covalently closed continuous loops and have displayed great potencies as gene regulators^10^. According to their sequence construction and sources from the genome, the so far discovered circRNAs can be mainly classified into three categories, namely exonic circRNAs, intronic circRNAs (ciRNAs) and exon-intron circRNAs (EIciRNAs). Exonic circRNAs are mainly found in the cytoplasm to act as miRNA sponges^11^,^12^. ciRNAs and EIciRNAs are speculated as efficient regulators *in cis/trans* for their parental genes in the nucleus^13^,^14^. Some circRNAs may also regulate gene expression by competitive alternative splicing^15^ or by acting as translation templates^16^. All these circRNA categories take active part in gene expression regulation, which also seem to be involved in the exquisite regulation network of spermatogenesis. But to date, little is known about the expression and functions of circRNAs in spermatogenesis.

Meanwhile, circRNAs in body fluid may also possess great potential in serving as novel biomarkers for various diseases, which falls into the realm of the increasingly heated concept of “liquid biopsy” in recent years. Liquid biopsy holds significant promise as a surrogate for traditional tissue biopsy because it is less invasive and could provide a comprehensive and representative view of the target tissues^17^. With their circular form and therefore resistance to exonucleolytic activities property, circRNAs could be stably detected in human body fluid, which endow them great potential in serving as liquid biopsy tools for various diseases. Scientists have found thousands of kinds of circRNAs in human blood^18^ and hundreds of types of them in human saliva^19^. These circRNAs are stable, abundant and specifically expressed in these body fluids, and thus may be developed as novel biomarkers for certain diseases^20^,^21^.

Although circRNAs were not previously reported in seminal plasma, we presumed circRNAs might also serve as biomarkers for male reproductive diseases based on our previous researches in seminal cell-free RNAs (cfs-RNAs)^22^,^23^,^24^,^25^,^26^,^27^. Seminal cell-free RNAs like cell-free seminal mRNAs (cfs-mRNAs) and cell-free seminal miRNAs (cfs-miRNAs) can be stably detected in seminal plasma and can comprehensively convey pathophysiological information from bilateral male reproductive organs (testes, epididymis, seminal vehicles and prostate glands). The levels of testis-derived cfs-RNAs should partly present their amounts in testis, thus may provide good reference to predict their expression and function in different pathological and physiological conditions of spermatogenesis. Current studies have revealed that circRNA is very stable among its noncoding RNA counterparts^28^, making it a seemingly better candidate in liquid biopsy for various diseases. In addition, the study of circRNAs in seminal plasma is quite necessary though there has been profiles of circRNAs in blood published. The existence of blood-testis barrier and blood-epididymis barrier may endow them distinct expression patterns from RNAs in the circulation. This has partly been confirmed in research of circRNAs in brain, which showed that the expression of circRNAs in cerebrospinal fluid is rather different from that in brain tissue due to the existence of the blood–brain barrier^29^.

Here, using the next-generation deep sequencing, we revealed the profile of circRNAs in normal human testis for the first time. The sequence feature, chromosome location, alternative splicing and other characteristics of the circRNAs in human testis were also studied. Some of these testis-derived circRNAs were randomly chosen for validation and their stability property and conformation were investigated in seminal plasma. Testis-derived circRNAs may take part in the post-transcriptional regulation of spermatogenesis and may be developed as novel non-invasive biomarkers for male reproductive diseases in the future.

## Results

### General characteristics of circRNAs in human testis

**Figure 1:**
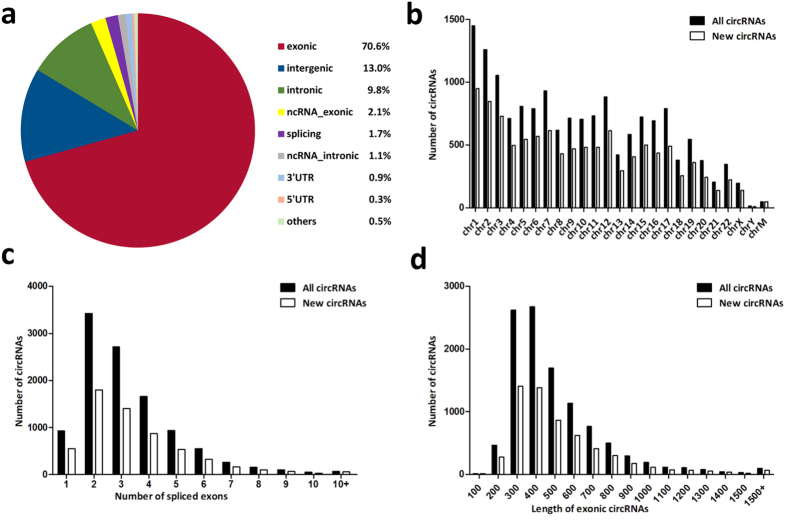
General characteristics of circRNAs in human testis. (**a**) Distribution of genomic regions that 15,996 circRNA derived from: exonic, intergenic, intronic regions, etc. (**b**) Chromosomal distribution of 15,996 circRNAs and 10,792 new discovered circRNAs. “chrM” represents mitochondrial genome. (**c**) Number of back-spliced exons of all circRNAs and new circRNAs. Most exonic circRNAs (about 90%) were comprised of five or less exons. (**d**) Distribution of exonic circRNA lengths of all circRNAs and new circRNAs. circRNAs no more than 1000 nt took up the majority (95%). The median length was 400 nt.

A total of 15,996 circRNAs were identified in human testis, by at least one read spanning a head-to-tail splice junction. Among them 5,204 were already included in circBase and 10,792 were first observed in this study. Based on their location on the genome, the host genes of these 15,996 circRNAs were derived from exonic regions (70.6%), intergenic regions (13.0%), intronic regions (9.8%) (Fig. 1a), etc.

According to their host gene location, the 15,996 circRNAs and 10,792 new circRNAs were widely scattered on all chromosomes, including the mitochondrial genome (Fig. 1b). Chromosomes 1–3 produced the most circRNAs (500 to 1000). Most other chromosomes generated hundreds of circRNAs, except chromosome Y and the mitochondrial genome (1,648 for all circRNAs and 948 for new circRNAs respectively).

Most exonic circRNAs, both novel and previously described, were comprised of no more than ten exons, among which five or fewer exons were the majority (about 90%) and circRNAs contained two or three exons were the most (Fig. 1c). The lengths of most exonic circRNAs (about 95%) were no more than 1,000 nucleotides (nt), and the median length was ~400 nt (Fig. 1d). All these results are mostly consistent with previous findings from other tissues and cells^30^,^31^,^32^, suggesting certain lengths are preferred in exon circularizing, probably for formation of more stable circles.

### Feature and function analysis of circRNAs and their host genes

**Figure 2:**
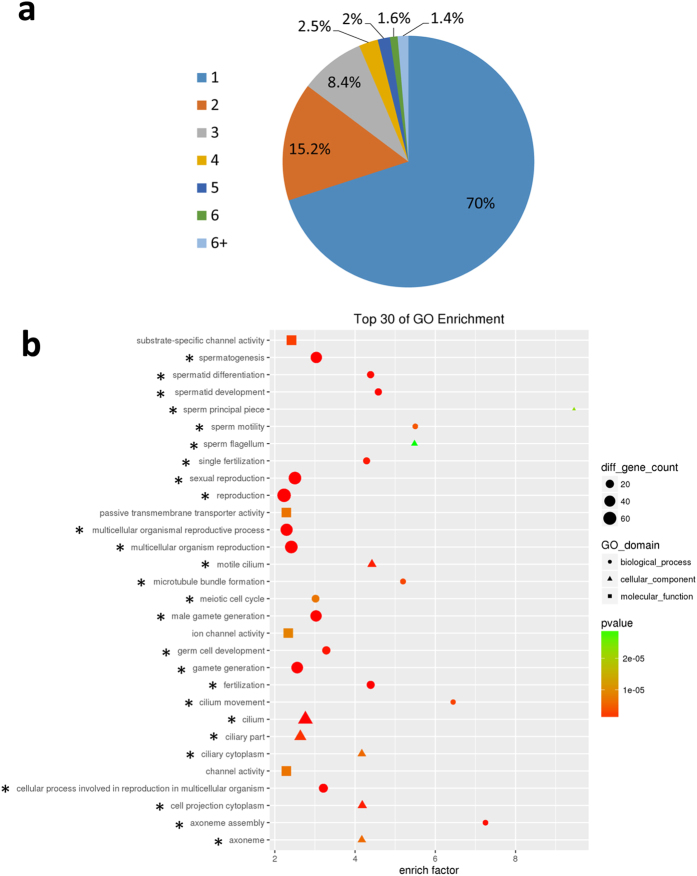
Feature and function analysis of 1017 first discovered circularizing genes. (**a**) Number of circRNAs produced from one gene (1,633 circRNAs from 1,017 host genes). 1, 2, 3 … represent the number of circRNAs per gene. (**b**) GO annotation of 1017 host genes. Top 30 of GO enrichment are shown here. *GO domains directly related with reproduction.

In the 15,996 circRNAs, 1,963 were not found with corresponding host genes. The rest 14,033 (87.7%) circRNAs were mapped to 5,928 host genes. Among them, 1,017 genes are first reported in this study to form circRNAs. Seventy percent of these 1,017 genes produced only one circRNAs and 94.6% generated no more than six (Fig. 2a). Only three genes could give rise to ten circRNAs or above (“circRNA hotspots”). In Gene Ontology (GO) analysis, the 1,017 first reported circularizing genes are closely related to reproduction domains such as spermatogenesis, sperm motility, meiotic cell cycle and fertilization, etc. In the top 30 of GO enrichment, 26 were directly related with reproduction (Fig. 2b). The remaining 4,911 genes show no such close relationship to reproduction in GO analysis, with only two directly related domains out of 30 top enriched GO terms (Fig. S1).

### Validation of predicted circRNAs in human testis

To validate these predicted circRNAs, 55 circRNAs of different abundance and lengths were randomly chosen for subsequent RT-PCR validation. They are all exonic circRNAs except one EIciRNA (circHIST1H2BA chr6: 25727079-25727268). Divergent primers were designed for circRNA detection (Tables S2, S3, and S4). Among the 55 circRNAs, 30 of them could be specifically amplified (Table S1). Nine could be amplified but was accompanied with other bands, which may result from nonspecific amplification or false positive detection. The rest could not be detected, most likely due to primer designing errors or other reasons. Of the 30 circRNAs, 16 were derived from generally expressed genes and 14 were testis-specific. Figure S2 presents 20 of them (10 generally expressed and 10 testis-specific). Sanger sequencing confirmed the predicted head-to-tail splice junctions (Fig. S3). The seven host genes of ten testis specific circRNAs are *LRWD1, STK31, HIST1H2BA, RNF17, BRDT, SMC1B* and *SPATA16*. In GO analysis, these genes are closely related to DNA replication^33^, spermatogonia development^34^, histone displacement^35^, androgen receptor interaction^36^, meiotic cell cycle^37^, male meiosis^38^ and spermatogenesis^39^ respectively.

### Circular characteristics of these testis derived circRNAs

**Figure 3:**
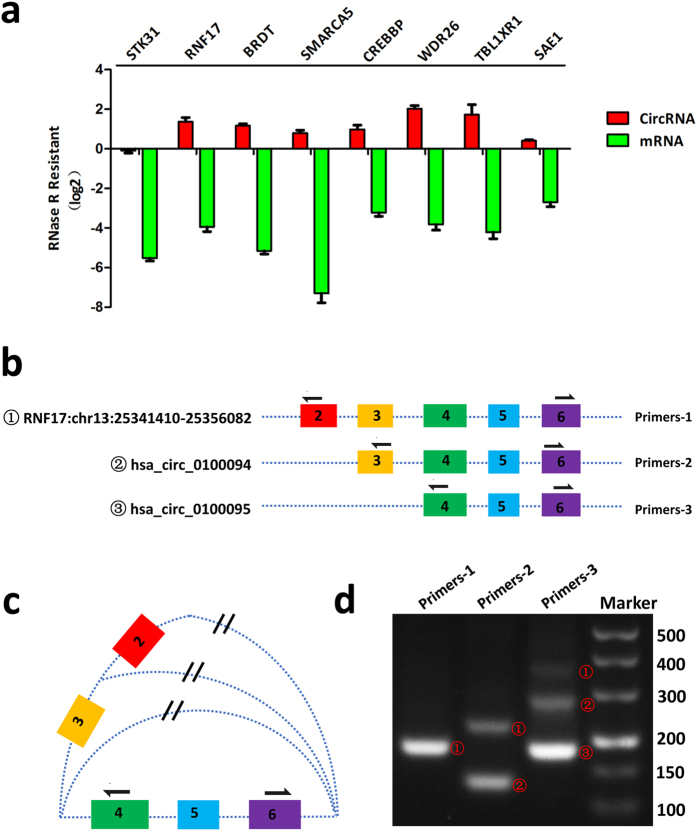
RNase R resistance and alternative splicing of circRNAs. (**a**) circRNAs were resistant to RNase R digestion compared with their linear isoforms. Total RNA was treated with RNase R or a mock treatment. The fraction of linear and circular isoforms was normalized to the value measured in the mock treatment respectively. (**b**) Different patterns of circularization for RNF17. The coloured blocks represent different exons of *RNF17.* Opened loop of three circRNAs and their corresponding amplification primer locations are shown here. (**c**) Primers set at exon 4 and exon 6 can amplify three kinds of circRNAs. ^**//**^The backsplice site of circRNA. (**d**) Electrophoretogram for three kinds of circRNAs derived from *RNF17* using different primers in RT-PCR.

RNase R can digest linear RNAs while leaving their circular isoforms intact. With their circular form, these testis derived circRNAs showed resistance to RNase R digestion compared to their corresponding mRNAs (Fig. 3a). After RNase R digestion, the expression of eight circRNAs did not obviously decrease but most of them increased notably after the treatment. We speculate the linear RNA depletion increase the relative amount of random primers and dNTPs for circRNAs in the reaction system, thus the amplification efficiency was enhanced. RNase R digestion is essential in the detection of circRNAs for it not only relatively increases the concentration but also ensures the purity of circRNAs, especially when some mRNA fragments are part of the circRNA loop.

We also observed alternative circularization phenomenon in these testis derived circRNAs. Alternative circularity usually occurs when different exons come to form circRNAs in two or more ways. Take the host gene *RNF17* for example. There were three types of circRNAs derived from this gene in our sequencing, namely circRNF17:chr13:25341410-25356082 (comprised of exon2-exon6), hsa_circ_0100094 (exon3-exon6), hsa_circ_0100095 (exon4-exon6). If we designed our forward primer at exon6 and reverse primer at exon2, the product was only the first circRNA. If primers were set at exon6 and exon3, the first and second circRNAs were detected. And the primers set at exon4 and exon6 amplified all three kinds of circRNAs (Fig. 3b,c and d, and Table S3). We also observed alternative splicing phenomenon in circRNAs derived from *STK31* and *SAE1* (Figs S5, S6).

### Testis derived circRNAs can be stably detected in seminal plasma

**Figure 4:**
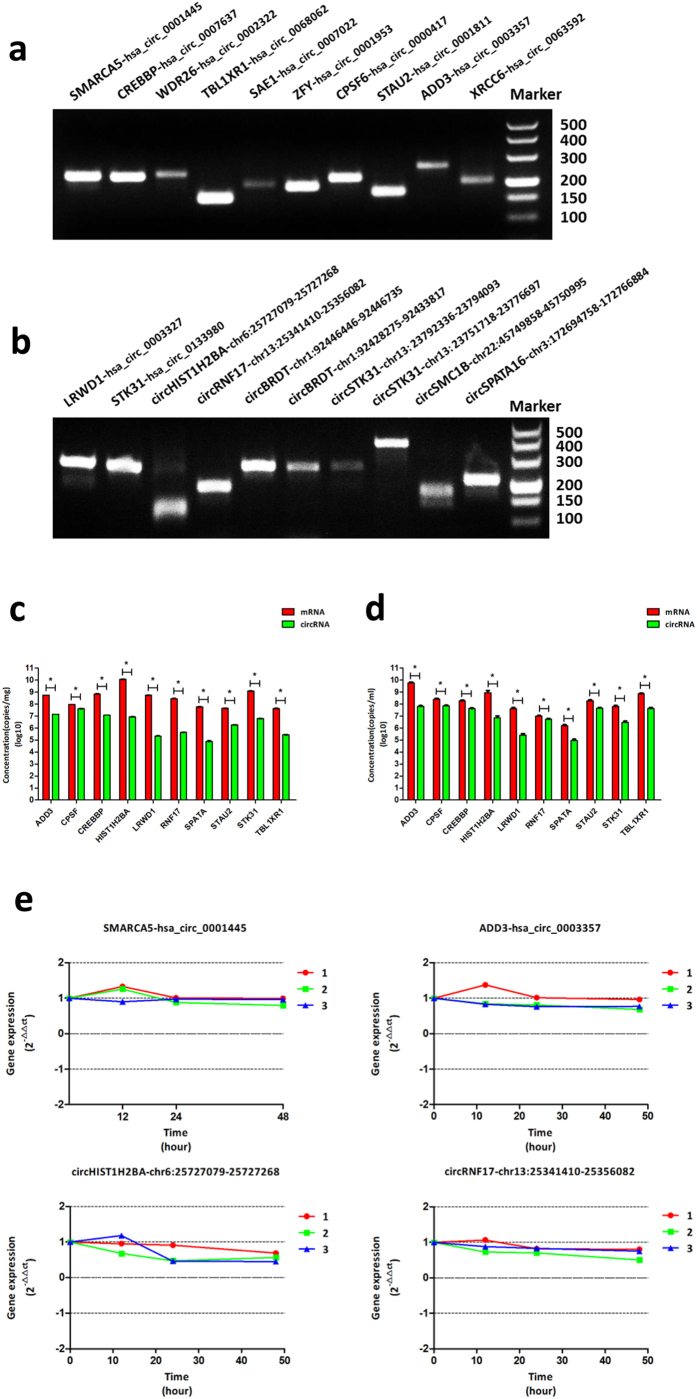
Testis-derived cell-free seminal circRNAs (cfs-circRNAs). (**a**) Detection of ten generally expressed cfs-circRNAs. (**b**) Ten testis specific cfs-circRNAs. (**c**) Absolute quantification of circRNAs and their corresponding mRNAs in testis tissue (copies/mg). circRNAs are less abundant than their mRNA counterparts in testis. The trends are similar in seminal plasma. *P < 0.05 (**d**) Absolute quantification of circRNAs and mRNAs expression in seminal plasma (copies/ml).The expression of the two isoforms in seminal plasma showed similar results with that in testis tissue. *P < 0.05 (**e**) The stability assay of four representative testis-derived cfs-circRNAs. cfs-circRNAs were rather stable at room temperature within 24 hours.

Next, the expression of these testis-derived circRNAs in seminal plasma was investigated. We chose 20 circRNAs that had been validated above as representative. Of them 10 were generally expressed and 10 were testis-specific. RT-PCR showed that all of the 20 circRNAs could be detected in seminal plasma (Fig. 4a,b). Absolute qPCR revealed that circRNAs were less abundant than mRNAs both in testis and in seminal plasma (Fig. 4c,d). However, our study found that these testis-derived circRNAs are rather stable in seminal plasma. Seminal plasma samples from three healthy volunteers were collected and incubated at room temperature for 0 h, 12 h and 24 h. RT-PCR was performed to analyze the expression change of these circRNAs. The expression levels of circRNAs were first standardized by internal reference ACTB. Then we set the values of 0 h as 1 and other values were recalculated according to the 0 h baseline. The results revealed that the expression of circRNAs showed no significant changes in the three time points. Four circRNAs are chosen to present the stability assay here (Fig. 4e). cfs-circRNAs hardly degraded in room temperature within 24 h after collection.

### A large proportion of testis derived circRNAs may be bound with proteins in seminal plasma

**Figure 5:**
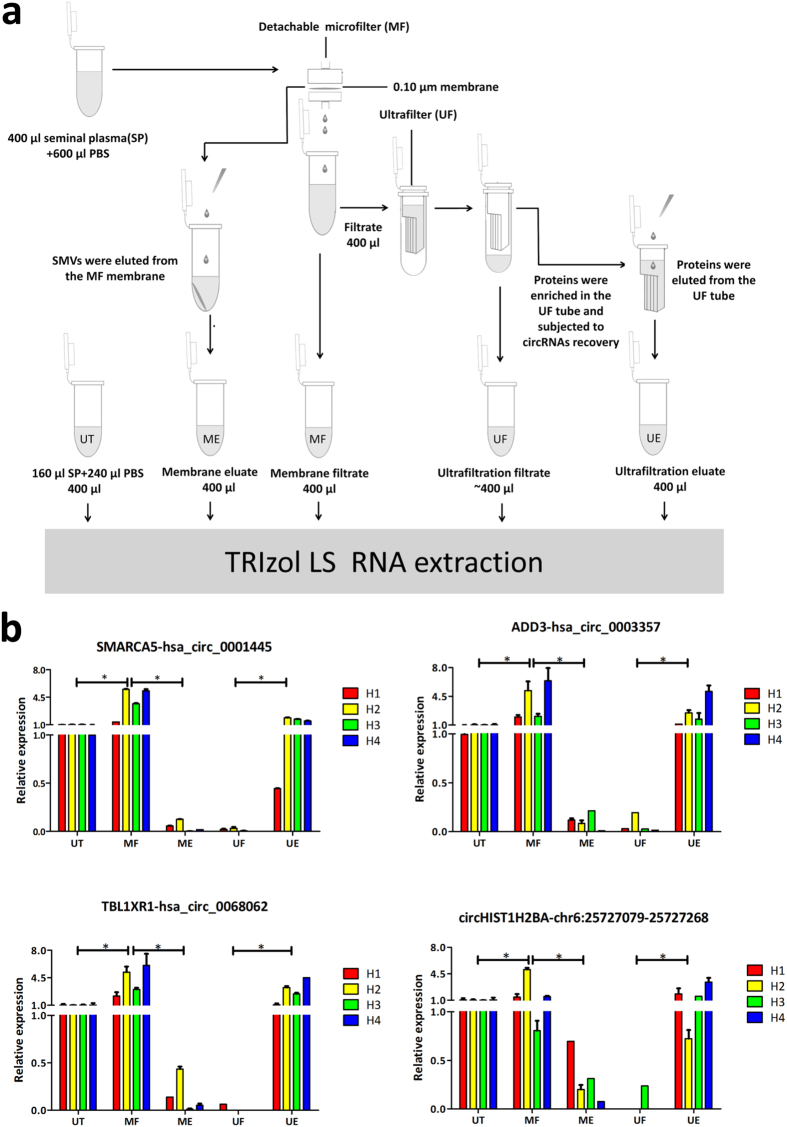
The conformation analysis of cell-free seminal circRNAs (cfs-circRNAs). (**a**) Workflow for harvesting seminal microvesicles (SMVs) and proteins for cfs-circRNA recovery. SMVs and proteins were eluted from the MF membrane and the UF tube by pipetting up and down, and then subjected to cfs-circRNA recovery. (**b**) Quantitive analysis of cfs-circRNAs in different components. UT: untreated seminal plasma; MF: membrane filtrate; ME: membrane eluate; UF: ultrafiltration filtrate; UE: ultrafiltration eluate. *P < 0.008 (post hoc Mann-Whitney U test with adjusted *P’* value of 0.008 according to the multiple comparison times of four) was considered as statistically significant in post hoc comparisons.

Considering their fairly stable characteristics in seminal plasma, we sought to explore the possible conformation of cfs-circRNAs. Seminal plasma was first passed through a 0.1 μm pore microfilter and then a 30 kDa concentration tube (Fig. 5a). Most of the circRNAs passed the 0.1 μm pore microfilter and retained in the 30 kDa concentration tube (Fig. 5b). The 0.1 μm pore size microfilter can retain particles larger than 0.1 μm, which is the size of a large proportion of seminal microvesicles (SMVs)^28^,^40^,^41^,^42^. And the 30 kDa ultrafilter can trap macromolecules such as protein complexes. We may preliminarily conclude that a large proportion of cfs-circRNAs may be present in the seminal plasma in the form of protein complexes. Meanwhile, a slight elevation in circRNAs expression after the 0.1 μm pore size filtration was observed. We infer this may be because the filtration depleted the cell debris and other impurities in the seminal plasma and increased the extraction efficiency of the cfs-circRNAs.

## Discussion

Our study for the first time profiled the circRNA expression pattern in human testis and analyzed their existing form in seminal plasma. circRNAs in human testis showed some similar but also distinct features with circRNAs from other tissues. And these circRNAs may participate in spermatogenesis for their host genes are closely related to this process. In addition, the study found that a large proportion of testis derived circRNAs could be stably detected in seminal plasma and most probably existed in the form of protein-complexes. They may be developed as novel biomarkers for male reproductive diseases.

Up till now, many circRNAs have been found in various normal human tissues and diseased tissues^30^. Researchers have identified 65,731 circRNAs in normal human brain^43^, 8,045 in heart^30^ and 3,982 in liver^30^. In our study, we predicted 15,996 circRNAs in normal human testis with 10,792 novel ones that had not been included in circBase before. Together with previous calculations^30^, there have been in total 159,493 unique human circRNA candidates discovered so far, making circRNAs probably the largest RNA families in human transcription. Compared to other organs, the expression of circRNAs in human testis is abundant, with numbers only secondary to that of brain. This is consistent with findings from the study of circRNAs profiles of different mouse tissues. They also found that brain and testis were the top two to generate circRNAs among all other mouse organs^44^. We speculate this is due to the need of massive and accurate gene expression regulations in spermatogenesis, which shares some similar features with that of the brain.

A vast majority of circRNAs were derived from exonic regions and intergenic regions, while others had reported that exonic regions and 5′ UTR sequences produced the most circRNAs^10^,^43^. Intergenic circRNAs are generated by intergenic regions between protein coding genes. Recently, intergenic noncoding RNAs such as intergenic lncRNAs have been suggested to participate actively in spermatogenesis^45^. Unlike other studies, our results revealed that intergenic circRNAs ranked second only to exonic circRNAs, indicating that the “dark matters” —gene intergenic regions may participate actively in gamete generation.

In our study, 1,017 host genes are first reported to form circRNAs. In GO analysis, these genes are involved with processes like spermatogenesis, sperm motility, meiotic cell cycle, fertilization and so on. Fourteen out of the 30 validated circRNAs are testis-specified. Their corresponding eight host genes are *BRDT, SMC1B, HIST1H2BA, LRWD1, STK31, RNF17, SMC1B and SPATA16*. These genes are also closely related to spermatogenesis. Since some exonic circRNAs were reported to act as sponges for miRNAs that target at its host gene in cytoplasm^46^ and some EIciRNAs were found to be efficient regulators *in cis* for their host gene in the nucleus^14^, these circRNAs may participate in spermatogenesis by interacting with their host genes. The study of these genes and their derivative circRNAs may provide new perspectives for the study of spermatogenesis. Generally, most circRNAs are less abundant than their corresponding mRNAs from the same host gene^9^,^47^,^48^,^49^. Only in rather limited tissues and under certain circumstances, the relative ratio was the contrary^44^,^47^. Researchers have found that the abundance of the circles was roughly 5–10% that of their linear counterparts^14^,^47^. In our experiment, we also revealed that circRNAs are less abundant than their corresponding mRNA for both generally expressed genes and testis specific genes. Alternative splicing is a common phenomenon in eukaryotes to ensure the diversity of coding proteins and is also observed in circRNAs. The alternative splicing of circRNAs is closely related to reverse complementary matches (RCM)—the flanking sequences that mediate the circularization of circRNAs^50^,^51^. Different combinations of RCMs produce different circRNAs from the same gene. Regulating factors like proteins may participate in the circularization process to enable the spatial and temporal-specific expression of certain circRNAs^52^. Though alternative splicing ensures the diversity of circRNAs, it also makes it difficult to specifically amplify a certain circRNA when their isoforms are present in the same sample. This is mainly due to their distinct circular forms that make it difficult to design specific primers to amplify certain circRNAs, especially when the target circRNA sequence is part of their larger loop counterparts.

Another promising application for the field of circRNAs is to be developed as novel biomarkers for various diseases. Seminal plasma contains abundant cell-free DNAs and RNAs and is an ideal non-invasive diagnosis tool in liquid biopsy for many male reproductive diseases as has been revealed by our previous studies^22^,^23^,^24^,^25^,^27^. For example, cfs-mRNAs were found to indicate the presence of germ cells in testis and the obstruction location of obstructive azoospermia, which provided valuable noninvasive information in the clinical decision of sperm retrieval and spermatogenesis promotion treatment^25^.

Like other cell-free seminal RNAs in seminal plasma, cfs-circRNAs also hold great promise to be developed as novel biomarkers for male infertile diseases. circRNAs have recently been found as active participant in gene regulation and our human testis circRNAs profiling study has revealed their underlying involvement with spermatogenesis. In the current study, we chose 20 testis-derived circRNAs as representative and found they all stably expressed in seminal plasma. Among them 10 were expressed specifically in testis. In GO analysis, their host genes are closely related to spermatogenesis process such as spermatogonia development^34^, meiotic cell cycle^37^, histone displacement^35^, androgen receptor interaction^36^, etc. Since the levels of testis-derived cfs-circRNAs should partly reflect their expression and function in testis like other cell-free nucleic acids do, cfs-circRNAs may convey some valuable information relating to sperm production and maturation in testis.

Stability is another important factor in evaluating the potential of a candidate biomarker. Though circRNAs are found to be less abundant than mRNAs both in testis and seminal plasma, we found cfs-circRNAs remained stable for 24 h in room temperature incubation. This property to some extent ensures their future application in clinical use.

With its circular form, the escape from RNase R digestion is a good but not sufficient protective factor to prevent cfs-circRNAs from degeneration, for there are many other types of RNase in seminal plasma. Therefore, we presumed there might be some unique protection mechanism for cfs-circRNAs. Enlightened by our previous findings that cfs-mRNAs were encapsulated in SMVs and cfs-miRNAs were bound with proteins^27^, we conducted a sequential multi-step centrifugation experiment and found that a large proportion of cfs-circRNAs may be bound with proteins in seminal plasma. It has been reported that some proteins can sequester and stabilize RNAs under certain circumstances^53^,^54^,^55^, so this particular protein-complex form of cfs-circRNAs may contribute to their stability in seminal plasma. In addition, this unique form also adds to the convenience of their extraction in seminal plasma. RNA extraction from seminal plasma is rather challenging due to the affluence in proteins, DNAs and polysaccharides of seminal plasma. But with the now existing many methods for protein fractionation and concentration, cfs-circRNAs may be extracted more easily due to their protein-complex form.

In conclusion, our study is the first to delineate the expression profile of circRNAs in human testis and their presence and characterization in seminal plasma. Abundant circRNAs were found to exist in human testis and the newly discovered 1,017 circularizing host genes are closely related to spermatogenesis. The sequence feature, chromosome location, alternative splicing and other characteristics of the circRNAs in human testis were also revealed, laying the basis for future functional studies of circRNAs in human testis. Many of these testis-derived cfs-circRNAs convey valuable information of spermatogenesis. Their most probable protein complex form may contribute to their stability in seminal plasma and provides much convenience in extraction of these cfs-circRNAs. All these endow them great prospects to be developed as novel noninvasive biomarkers for male infertile diseases in the future.

## Methods

### Sample preparation

The study protocol was approved by the ethic committee of Tongji Medical College, Huazhong University of Science and Technology. Informed consent was obtained from each subject. All these experiments were performed in accordance with the relevant guidelines and regulations.

Normal and intact human testis tissues were collected from a healthy middle-aged man (with normal function of spermatogenesis) during the condition of a testicle injury. The tissues were immersed in dry ice for transportation after dissection and were immediately preserved in liquid nitrogen until use.

Semen samples were gathered from healthy volunteers after 3–5 days of sexual abstinence and all met the criteria for normal semen from WHO Laboratory Manual for the Examination and Processing of Human Semen (5^th^ edition). After being liquefied at 37 °C for 30 min, the semen samples were subjected to a two-step centrifugation to harvest cell-free seminal plasma. First we centrifuged the semen at 1,600 g for 10 min at 4 °C to remove sperm. Then the supernatant was centrifuged at 16,000 g for another 10 min at 4 °C to remove cell debris. We collected the supernatant for subsequent analysis.

### RNA isolation and RNase R treatment

Total RNA was extracted from testis tissue using TRIzol (Invitrogen, NY) according to the manufacturer’s protocol. For deep sequencing, the sample was depleted of ribosomal RNAs and then linear RNAs by RNase R (RNR07250, Epicenter). Total RNA from seminal plasma was isolated with TRIzol LS (Invitrogen, NY) with similar procedures.

### cDNA library preparation and sequencing

cDNA library was generated according to the TruSeq library preparation protocol (Illumina). Sequencing was performed on Illumina HiSeq 3000 platform in PE150 sequencing mode. Sequencing data are publicly available in SRA database under accession number SRX2254041.

### Sequence mapping and circRNA annotation

Sequence reads were first multiply mapped using TopHat against the GRCh37/hg19 human reference genome with the UCSC Genes annotation. Unmapped reads were then extracted and mapped onto the relevant reference genome using TopHat-Fusion to detect the “fusion junctions”. The computational pipeline CIRCexplorer was used to obtain back-spliced junction reads for circular RNA prediction^10^. ANNOVAR was used to analyze the functional elements (exon, intron, 5′UTR, etc.) from which the circRNAs were derived.

### cDNA synthesis, PCR and qPCR

Total RNA was reverse transcribed using random primers with the RevertAid First Strand cDNA Synthesis Kit (Thermo Scientific) according to the manufacturer’s protocol. Primer sequences are shown in Tables S2, S3 and S4. qPCR reactions were carried out using SYBR Premix Ex Taq II (Takara, Dalian, China) on a Roche LightCycler96 thermocyler. PCR products were Sanger-sequenced directly using the amplification primers. Absolute quantification was based on calibration curves made with RT-PCR products. The PCR products were extracted from agarose gel with EZNA Gel Extraction Kit (Omega Bio-tek, Doraville, GA, USA). The DNA concentration (l g/ml) = Absorbance 260 × 50 × Dilution Factor, the copy concentration (copies/ml) = 6.02 × 10^23^ (copy/mol) × DNA concentration (g/ml)/DNA length (bp)/660 (g/mol/bp)^56^. The DNA was diluted serially in 10-fold increments to construct the calibration curves. And the copy concentration was calculated with correlative equation. Primer sequences for absolute qPCR are shown in Table S5.

### RNase R treatment

Total RNA of 5 ng was incubated with 20 U RNase R (Epicentre) in 1x RNase R buffer at 37 °C for 45 min, and then placed on ice. Mock treatment was the same but without addition of RNase R. cDNA synthesis and qPCR procedures were the same as previous described. qPCR Ct values were automatically calculated and ΔCt was termed as Ct (RNase R treatment)-Ct (mock treatment). The expression of circRNAs and mRNAs before the RNase R treatment were normalized as 1.2^−ΔCt^ was used to compare the expression of circRNAs and mRNAs after RNase R digestion.

### Seminal plasma circRNAs enrichment by microfilter and ultrafilter

The same volume of 400 μl was maintained in all experiments for RNA extraction. First, seminal plasma of 160 μl was filled up to 400 μl with 1 × PBS and labeled as “untreated” (UT). We took another 400 μl of seminal plasma and mixed with 600 μl of 1 × PBS. The 1,000 μl mixture was first passed through the 0.1 μm pore sized microfiltration membrane placed in a detachable microfilter. Approximately 900 μl filtrate was collected. We removed 400 μl to a new tube and labeled it as “the membrane filtrate” (MF). After filtration, we took the membrane out of the microfilter and eluted it with 1 × PBS by pipetting up and down 30 times in an Eppendorf tube. Then the eluate was transferred to a new tube and labeled as “membrane eluate” (ME). We then took another 400 μl from the 900 μl filtrate and placed it in ultrafilter for centrifugation at 15,000 g, 20 min. The filtrate from the ultrafilter (approximately 350–400 μl) was denoted as “ultrafiltration filtrate” (UF). Then the concentration tube was eluted with 400 μl 1 × PBS and the eluate was transferred to a new tube and named as “ultrafiltration eluate” (UE). The overall workflow chart was shown in Fig. 5a.

### Statistical analysis

Experiment values are presented as means ± SEM. Means of groups were analyzed by Kruskal-Wallis H test. *P*-value of less than 0.05 was considered as statistically significant. Post hoc comparisons were carried out using Mann-Whitney U test, with adjusted *P’* value of 0.008 according to the multiple comparison times (n = 4 in the conformation analysis).

## Additional Information

**How to cite this article**: Dong, W.-W. *et al*. Identification and characterization of human testis derived circular RNAs and their existence in seminal plasma. *Sci. Rep.* **6**, 39080; doi: 10.1038/srep39080 (2016).

**Publisher's note:** Springer Nature remains neutral with regard to jurisdictional claims in published maps and institutional affiliations.

## Supplementary Material

Supplementary Information
