# Supplementary material for: Identification and characterization of human testis derived circular RNAs and their existence in seminal plasma
Source: Sci Rep. 2016 Dec 13;6:39080. doi: 10.1038/srep39080 (PMC5153637; doi:10.1038/srep39080)
Supplement: Supplementary Information [file srep39080-s1.pdf]

Identification and characterization of human testis derived circular RNAs and their existence in seminal plasma

Wei-Wei Dong[1, +], Hui-Min Li[1, +], Xing-Rong Qing[1], Dong-Hui Huang[1,2], Hong-Gang Li[1, 2,*]

[1]Family Planning Research Institute/Center of Reproductive Medicine, Tongji Medical College, Huazhong University of Science and Technology, Wuhan, 430030, P. R. China

[2]Wuhan Tongji Reproductive Medicine Hospital, Wuhan, 430030, P. R. China

+These authors contribute equally to this work

* Correspondence to: PhD. Hong-Gang Li, Family Planning Research Institute, Tongji Medical College, Huazhong University of Science and Technology, Hangkong Road 13, Wuhan 430030, P. R. China. Tel.: +86 27 83692651; Fax.: +86 27 83692651; E-mail: lhgyx@hotmail.com.

Table S1. List of 30 validated testis derived circRNAs

**Gene Symbol**	**circRNA ID**	**Position**	**Strand**	**Genomic length （nt）**	**Spliced length （nt）**	**Exon Composition**	**Best transcript**	**Count**
SMARCA5	hsa_circ_0001445	chr4:144464662-144465125	+	464	269	15-16	NM_003601	662
CREBBP	hsa_circ_0007637	chr16:3900298-3901010	-	713	713	2	NM_004380	168
WDR26	hsa_circ_0002322	chr1:224605962-224612356	-	6395	392	4-6	NM_025160	15
TBL1XR1	hsa_circ_0068062	chr3:176782708-176816329	-	33622	179	2-3	NM_024665	7
SAE1	hsa_circ_0007022	chr19:47646751-47673180	+	26430	635	2-6	NR_027280	22
ZFY	hsa_circ_0001953	chrY:2821950-2829687	+	7738	662	2-3	NM_003411	30
CPSF6	hsa_circ_0000417	chr12:69644909-69656342	+	11434	1599	2-9	NM_007007	154
STAU2	hsa_circ_0001811	chr8:74585342-74601048	-	15707	296	3-4	NM_014393	23
ADD3	hsa_circ_0003357	chr10:111883775-111890244	+	6470	589	10-13	NM_016824	9
XRCC6	has_circ_0063592	chr22:4205907-42054356	+	1450	231	10-11	NM_001469	78
LRWD1[+]	hsa_circ_0003327	chr7:102106264-102106717	+	454	352	2-3	NM_152892	712
STK31[+]	hsa_circ_0133980	chr13: 23775175-23776697	+	1541	534	7-8	NM_001260504	1
HIST1H2BA[#+]	None	chr6:25727079-25727268	+	190	190	1[*]	NM_170610	12060
RNF17[+]	None	chr13:25341410-25356082	+	14673	481	2-6	NM_031277	13
BRDT[#+]	None	chr1:92446446-92446735	+	290	290	10	NM_001242810	10
BRDT[#+]	None	chr1:92428275-92433817	+	5543	482	3-5	NM_001242805	8
STK31[+]	None	chr13: 23792336-23794093	+	1758	276	9-10	NM_001260504	38

STK31+	None	chr13: 23751718-23776697	+	24980	967	2-8	NM_001260504	28
SMC1B#+	None	chr22:45749858-45750995	-	1138	312	20-21	NM_148674	3
SPATA16+	None	chr3:172694758-172766884	-	72127	321	3-5	NM_031955	8
SAE1	hsa_circ_0002444	chr19:47646751-47658470	+	11720	529	2-5	NR_027280	12
SAE1	hsa_circ_0051660	chr19:47653459-47658470	+	5012	417	3-5	NR_027280	2
SAE1	hsa_circ_0007219	chr19:47653459-47656297	+	2839	317	3-4	NR_027280	9
RNF17+	hsa_circ_0100094	chr13:25348951-25356082	+	7132	386	3-6	NM_031277	28
RNF17+	hsa_circ_0100095	chr13:25352433-25356082	+	3650	294	4-6	NM_031277	58
BRDT#+	None	chr1:92428275-92430321	+	2047	367	3-4	NM_001242805	5
ADD3	None	chr10:111878345-111886261	+	7917	1041	6-12	NM_016824	25
TEX14+	None	chr17:56690782-56693666	-	2885	369	7-9	NM_198393	35
ADAMTS13	hsa_circ_0089372	chr9:136302869-136303486	+	618	270	13-14	NM_139025	64
XRCC6	has_circ_0063591	chr22:42046727-42046895	+	169	169	8	NM_001469	120

* EIciRNAs (exon-intron circRNAs): comprised of exons and introns. # These genes are first discovered by us to form circRNAs (not included in CircBase). +These genes are testis-specific.

Table S2. Primers of 30 validated testis derived circRNAs for general and quantitive RT-PCR

Gene Symbol	circRNA ID or Position	Exon Composition	Forward primer	Reverse primer	Length （nt）	AT(℃)
SMARCA5	hsa_circ_0001445	15-16	F:TCTAGACTGCAGAGATGAATGAAAA	R:ATCTTCATCAGTGATCTCACTTTCC	241	62
CREBBP	hsa_circ_0007637	2	F:AGTCATGAATGGATCTCTTGGGG	R:CATTGGGTATCAGCTCATCAGGA	235	58
WDR26	hsa_circ_0002322	4-6	F:CCCCACGGCGTTTACAGA	R:TTGTATTTCAGCGGCGTCAA	240	58
TBL1XR1	hsa_circ_0068062	2-3	F:CCTGTGTTGTGACCTCATGGT	R:GCATTGATGTGGGGATGTGC	161	62
ZFY	hsa_circ_0001953	2-3	F:GTGCCCCTGAAGCAGTCATAG	R:GCATCAGCTCCTATTCCATCAAA	192	58
CPSF6	hsa_circ_0000417	2-9	F:TCCCGTAGTAGAGACCGTCA	R:CTCGGTCTTCTGGGGCATC	220	58
STAU2	hsa_circ_0001811	3-4	F:TGGAACTGAATGGGCTTGCT	R:CTGCCTTCGGATTCCCATGT	171	58
ADD3	hsa_circ_0003357	10-13	F:CCACCAGCTCCTCCTAACC	R:TTTTTCACGCTGTTGCCTCTG	259	62
XRCC6	has_circ_0063592	10-11	F:TGACAGCTTTGAGAACCCCG	R:TAGCCTTCATCTTGCCCACC	200	62
LRWD1^{+}	hsa_circ_0003327	2-3	F:ACGCTCCGTAAGGTCAATGG	R:ACTGGCACAAGGCAGTAACA	269	58
STK31^{+}	hsa_circ_0133980	7-8	F:AAGTAGAGCAGATTGCCCAGG	R:AGGAATGGGGCTTTTGAGGT	269	62
HIST1H2BA$^{\#+}$	chr6:25727079-25727268	1*	F:AAGGAAGGCAAAAAGCGCAA	R:CTCCGGCATAGCTACCACAC	129	62
BRDT$^{\#+}$	chr1:92446446-92446735	10	F:GAGCCTTCTCTGAGCAATTCCA	R:CGCCCAAGTTTATCTCCAGGG	265	62
BRDT$^{\#+}$	chr1:92428275-92433817	3-5	F:AGATGCCACAAGAAGAGCAAGT	R:CATCCACAGGACGTTGAAAGG	260	62
STK31^{+}	chr13: 23792336-23794093	9-10	F:AAGTGTTGGATGAAGGGTGCT	R:TTCCGAAACGGACACTGATGT	255	62
STK31^{+}	chr13: 23751718-23776697	2-8	F:CCATTCCTTTGTGGGGGCATA	R:TGACTTCCAACCACATCTTCCA	371	62
SMC1B$^{\#+}$	chr22:45749858-45750995	20-21	F:TTCACCCAGTGTTTTGAGCAT	R:GGGGCTGCTGTTTTCAGTAAG	173	58
SPATA16^{+}	chr3:172694758-172766884	3-5	F:GAGTGCCATGATTGCTGACT	R:GCAAGATCTGGTTTCCTCATCC	213	58

ADD3	chr10:111878345-111886261	6-12	F:ACACAAACCCGAATGAGGTACTA R:GATTGCAGCATGGGGACTGA	215	62
TEX14+	chr17:56690782-56693666	7-9	F:GAAAACCCGCCTTGTGTACG R:CCTGCTCCCATTCCACACTA	217	62
ADAMTS13	hsa_circ_0089372	13-14	F:ATGGACTCCCAGCAGGTATGG R:TTGGCATACACCGGGTCC	196	58
XRCC6	has_circ_0063591	8	F:TGGGTTTCAAGCCGTTGGTA R:TCAAACCTGGATCATCAAACCG	163	58
BRDT+	chr1:92428275-92430321	3-4	F:CAACGTCCTGTGGATGCTGTG R:TCGACTTGGCAGAGACATTCTT	229	62

The primers for circRNAs derived from *RNF17* and *SAE1* are shown in Table S3 and Table S4 respectively. * EIciRNAs (exon-intron circRNAs): comprised of exons and introns.

These genes are first discovered by us to form circRNAs (not included in circBase). + These genes are testis-specific.

Table S3. Primers for alternative circularized circRNA products of *RNF17*

**Primers**	**Primer sequences(5'-3')**	**Forward Primer**	**Reverse Primer**	**Aim products**	**Length (nt)**	**AT(℃)**
Primers-1	F: ACTTGAACACATGCAGAAGCA<br>R:CAGGGCATATAATTGTGGTGCATT	Exon 6	Exon 2	① circRNF17:chr13:25341410-25356082	186	62
Primers-2	F: TGCAGAAGCAAACGATAGAGGA<br>R: GCCATTGGGTAGTAGCGTTG	Exon 6	Exon 3	① circRNF17:chr13:25341410-25356082	227	58
				②hsa_circ_0100094	132	
Primers-3	F: CACTTGAACACATGCAGAAGCA<br>R:GTCTTGTCTGTGGAGGCTGAA	Exon 6	Exon 4	① circRNF17:chr13:25341410-25356082	371	58
				②hsa_circ_0100094	276	
				③hsa_circ_0100095	184	

Table S4. Primers for alternative circularized circRNA products of *SAE1*

Primers	Primer sequences(5'-3')	Forward Primer	Reverse Primer	Aim products	Length （nt）
Primers-1	F:TCTGCCCTGTTAAAGAAGCCC<br>R: GATCCAGCATGGTCAGTCCT	Exon 6	Exon 2	① hsa_circ_0007022	200
Primers-2	F: TGCCAAAGTTAGCCAAGGAGTA<br>R: AGTCCTTTCACTCCTGCCAAG	Exon 5	Exon 2	① hsa_circ_0007022	281
				② hsa_circ_0002444	175
Primers-3	F: GCCAAAGTTAGCCAAGGAGTAG<br>R: TCAGTGTCCACCTTCACATCC	Exon 5	Exon 3	① hsa_circ_0007022	430
				② hsa_circ_0002444	324
				③ hsa_circ_0051660	212
Primers-4	F: CTGCTCCAGGGATGTCATAGTT<br>R: TTCGGCCAACAGACCCAGTA	Exon 4	Exon 3	① hsa_circ_0007022	505
				② hsa_circ_0002444	399
				③ hsa_circ_0051660	287
				④ hsa_circ_0007219	187

Table S5. Primers of 10 circRNAs and mRNAs for absolute qRCR

Gene Symbol	Forward primer	Reverse primer	Length （nt）	AT (℃)
circADD3	F:CCACCAGCTCCTCCTAACC	R:TTTTTCACGCTGTTGCCTCTG	259	62
mADD3	F:TCAGTTGCTTGCTGGAATTG	R:ATGGGTTAGGAGGAGCTGGT	111	60
circCPSF6	F:TCCCGTAGTAGAGACCGTCA	R:CTCGGTCTTCTGGGGCATC	220	58
mCPSF6	F:CTTTCTTTCCTCCACCAAC	R: GCAGTATCCATTTCCCTTC	136	60
circCREBBP	F:AGTCATGAATGGATCTCTTGGGG	R:CATTGGGTATCAGCTCATCAGGA	235	58
mCREBBP	F:AGACCCTAACGCAGGTTTCC	R:GGAAGGTGGGCAAACTGTTG	210	58
circHIST1H2BA	F:AAGGAAGGCAAAAAGCGCAA	R:CTCCGGCATAGCTACCACAC	129	62
mHIST1H2BA	F:GCAAAAAGCGCAAGAGGACC	R:CGCTCGCTATACGCTCAAAG	150	62
circLRWD1	F:ACGCTCCGTAAGGTCAATGG	R:ACTGGCACAAGGCAGTAACA	269	58
mLRWD1	F:GTGCCCAACCAGGACTAC	R: ATTCCACTTCACACACCCT	190	60
circRNF17	F:ACTTGAACACATGCAGAAGCA	R:CAGGGCATATAATTGTGGTGCATT	186	62
mRNF17	F:ACACATGCAGAAGCAAACGA	R:GGCGATAATTGTAATGCTCTTGCT	234	62
circSPATA16^{+}	F:GAGTGCCATGATTGCTGACT	R:GCAAGATCTGGTTTCCTCATCC	213	58
mSPATA16	F:CACCCACCTAACATCCTG	R: TTTCTTCACCTTCTGCCTT	166	60
circSTAU2	F:TGGAACTGAATGGGCTTGCT	R:CTGCCTTCGGATTCCCATGT	171	58
mSTAU2	F:CAACTGTGGAACTGAATGG	R: ACTGGGCAATGATACCTCT	139	60
circSTK31	F:AAGTAGAGCAGATTGCCCAGG	R:AGGAATGGGGCTTTTGAGGT	269	62
mSTK31	F:GGTCTAACGTCAGCCTGGAA	R:TCAGTATTTCCATCTTAGCTGCCA	249	62
circTBL1XR1	F:CCTGTGTTGTGACCTCATGGT	R:GCATTGATGTGGGGATGTGC	161	62
mTBL1XR1	F:AGCAGTGATGAGGTCAACTTCTTG	R:GATGATAGAAATCAATGCAGCGGG	150	62

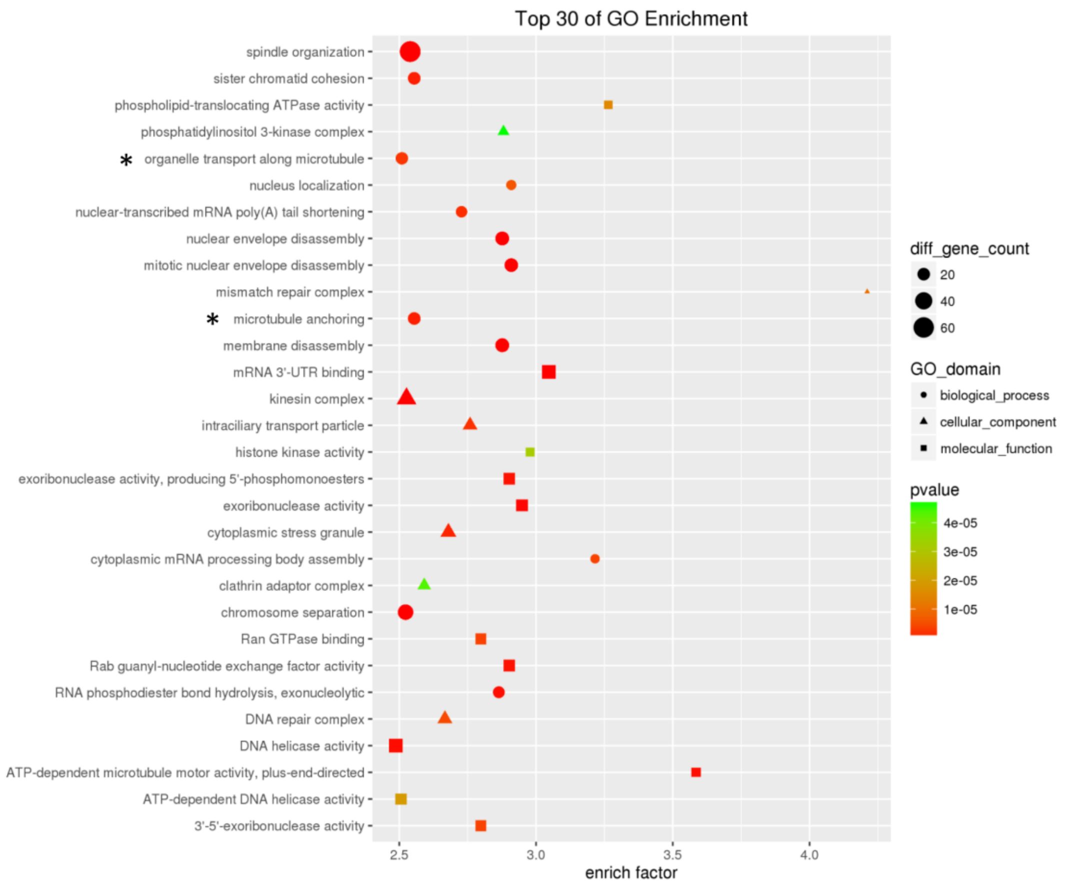

Figure S1. GO annotation of 4911 host genes (5928-1017) in human testis. Top 30 of GO enrichment are shown here. * GO domains directly related with reproduction.

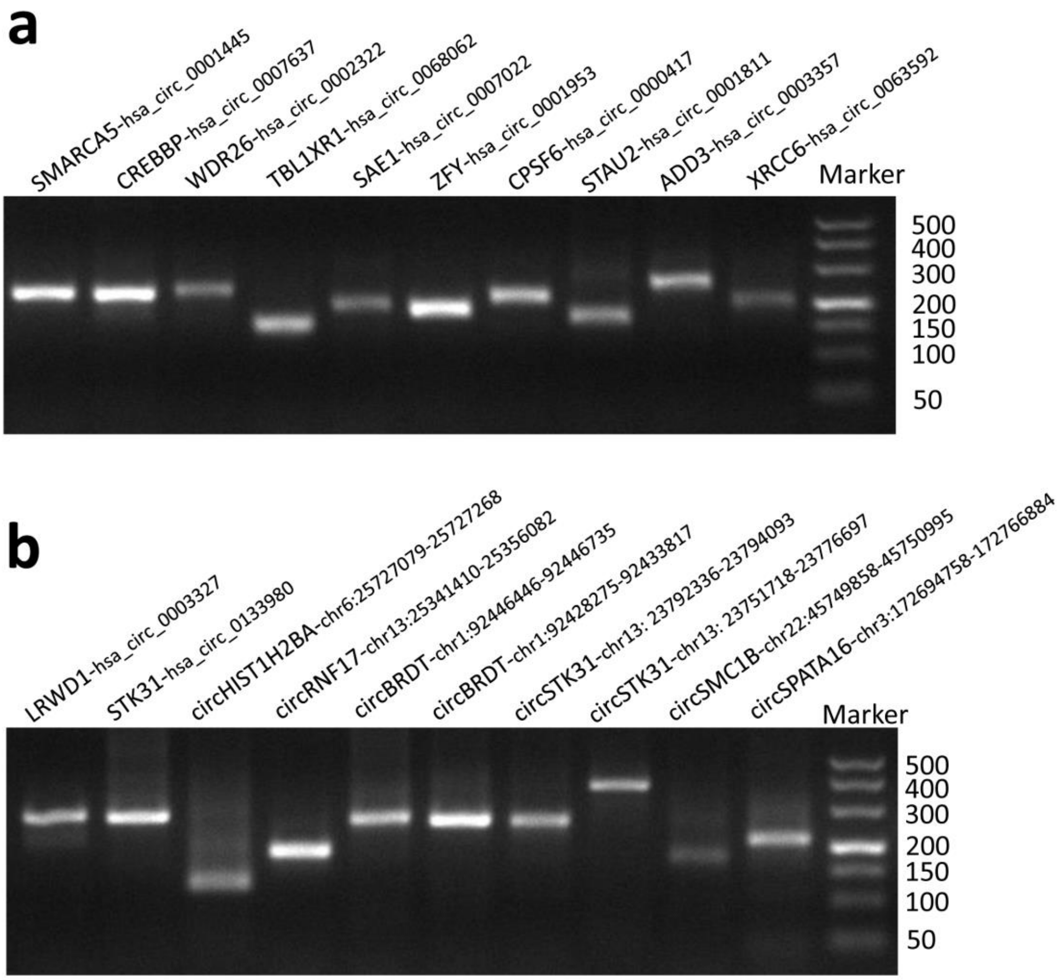

Figure S2. Twenty validated circRNAs in human testis. (a) circRNAs derived from commonly expressed host genes. (b) circRNAs from testis specific expressed genes.

a

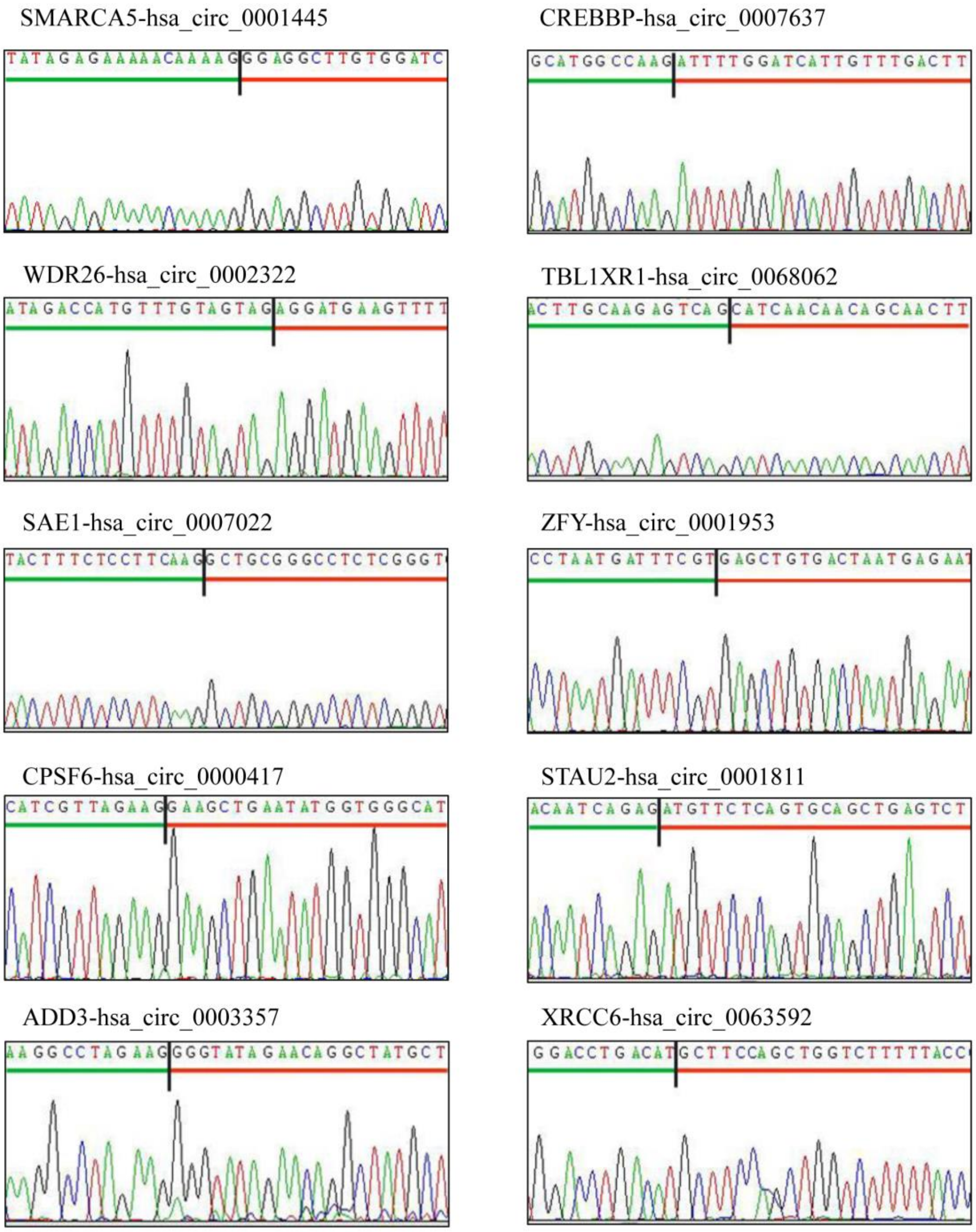
SMARCA5-hsa_circ_0001445
CREBBP-hsa_circ_0007637
WDR26-hsa_circ_0002322
TBL1XR1-hsa_circ_0068062
SAE1-hsa_circ_0007022
ZFY-hsa_circ_0001953
CPSF6-hsa_circ_0000417
STAU2-hsa_circ_0001811
ADD3-hsa_circ_0003357
XRCC6-hsa_circ_0063592

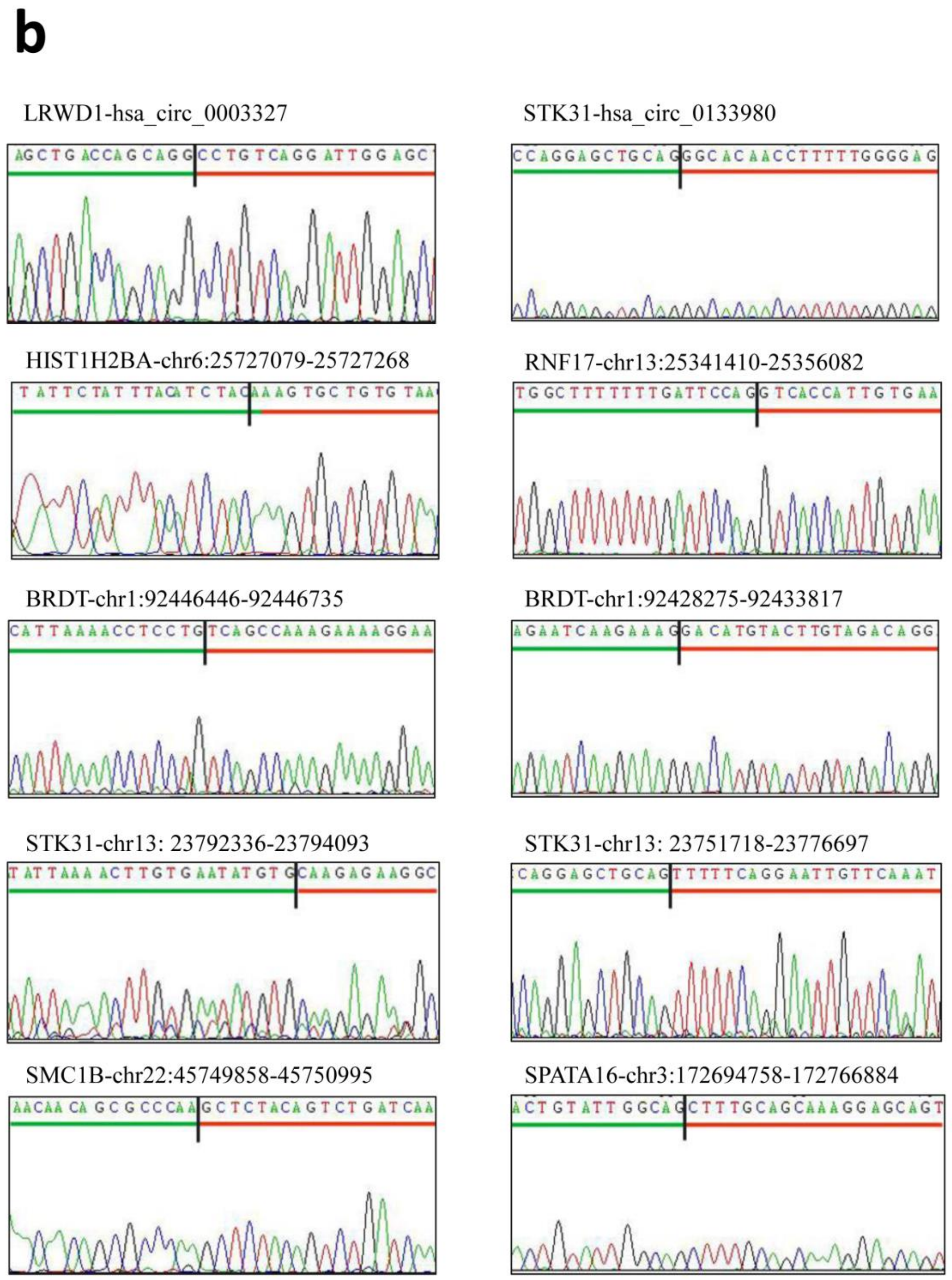

Figure S3. Sanger sequencing map for 20 of the 30 validated circRNAs. (a) circRNAs derived from commonly expressed host genes. (b) circRNAs from testis specific expressed genes. Black vertical line: splicing junction; green horizontal line: the 3 'end of the splice donor; red horizontal line: the 5 'end of the splicing receptor.

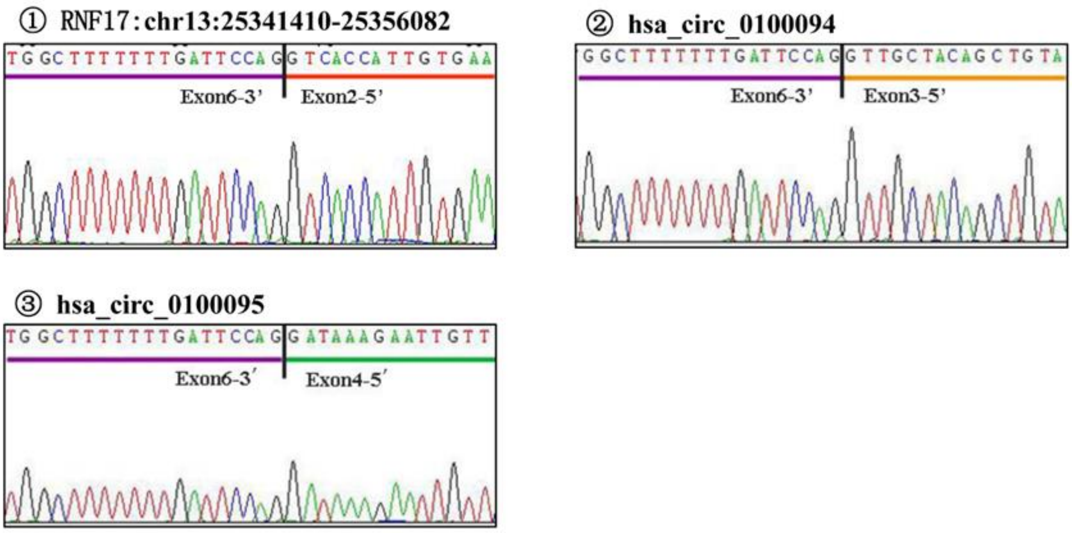

Figure S4. Sanger sequencing map for three alternative splicing products of *RNF17*.

Black vertical line: splicing junction; green horizontal line: the 3 'end of the splice donor; red horizontal line: the 5 'end of the splicing receptor.

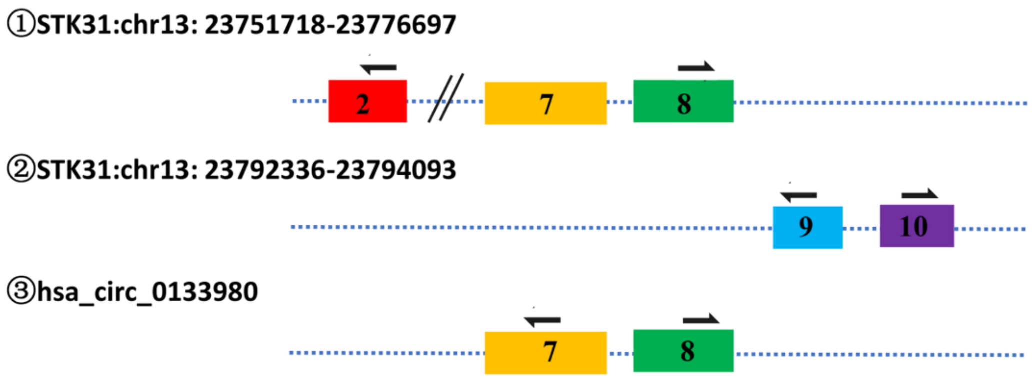

Figure S5. Alternative splicing of *STK31* generated different circRNAs. The coloured blocks represent different exons of *STK31*. Arrows show the primer location set as different exons.

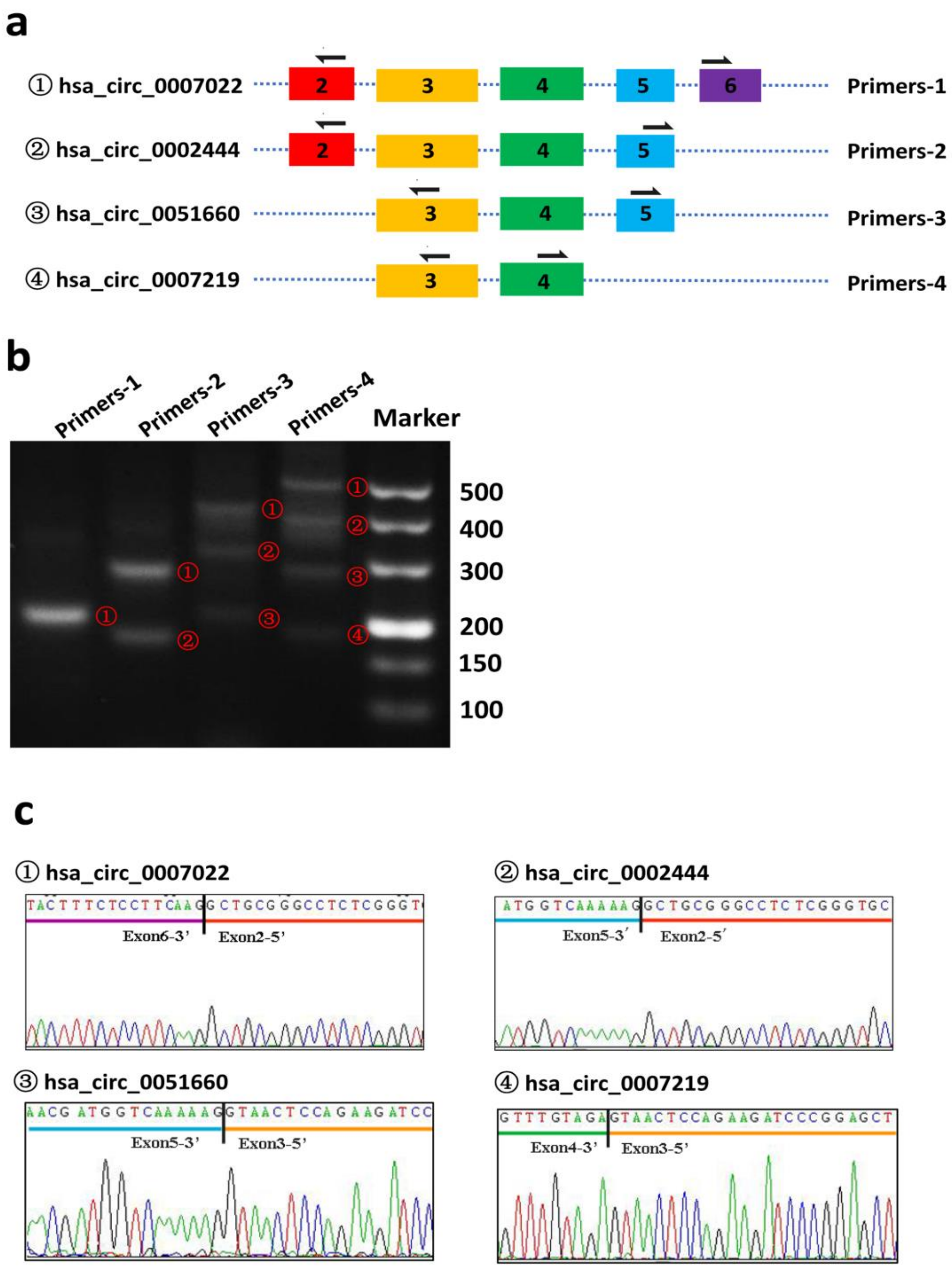

Figure S6. Alternative splicing of *SAE1* formed four kinds of circRNAs. (a) Mode pattern of different circularizing ways of *SAE1*. The coloured blocks represent different exons of *SAE1*. Arrows show the primer location set as different exons. (b) RT-PCR validation for three kinds of circRNAs derived from *SAE1* using different primers. (c) Sanger sequencing map for the three alternative splicing circular products of *SAE1*.

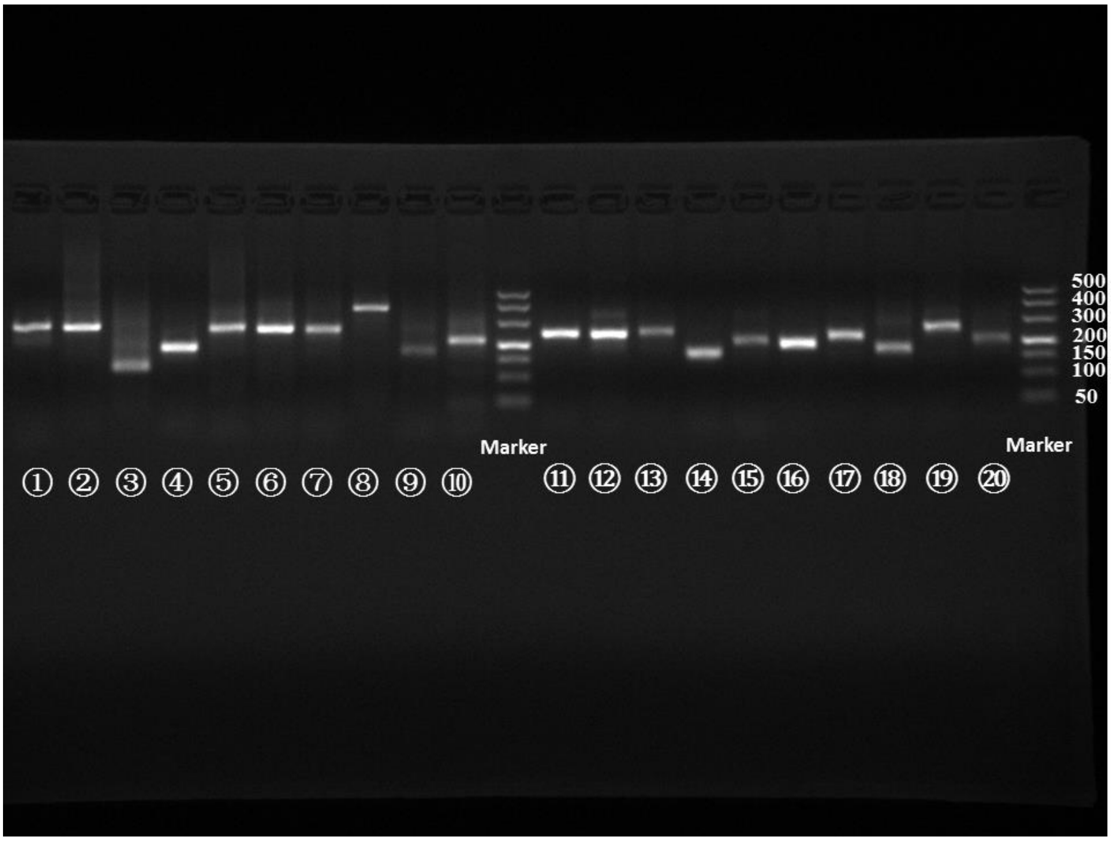

Figure S7. Twenty validated circRNAs in human testis (full-length gels and blots of Fig. S2).

① LRWD1-hsa_circ_0003327； ② STK31-hsa_circ_0133980；

③ circHIST1H2BA-chr6:25727079-25727268； ④ circRNF17-chr13:25341410-25356082；

⑤ circBRDT-chr1:92446446-92446735； ⑥ circBRDT-chr1:92428275-92433817；

⑦ circSTK31-chr13: 23792336-23794093； ⑧ circSTK31-chr13: 23751718-23776697；

⑨ circSMC1B-chr22:45749858-45750995； ⑩ circSPATA16-chr3:172694758-172766884;

⑪ SMARCA5-hsa_circ_0001445; ⑫ CREBBP-hsa_circ_0007637;

⑬ WDR26-hsa_circ_0002322; ⑭ TBL1XR1-hsa_circ_0068062;

⑮ SAE1-hsa_circ_0007022; ⑯ ZFY-hsa_circ_0001953;

⑰ CPSF6-hsa_circ_0000417; ⑱ STAU2-hsa_circ_0001811;

⑲ ADD3-hsa_circ_0003357; ⑳ XRCC6-hsa_circ_0063592

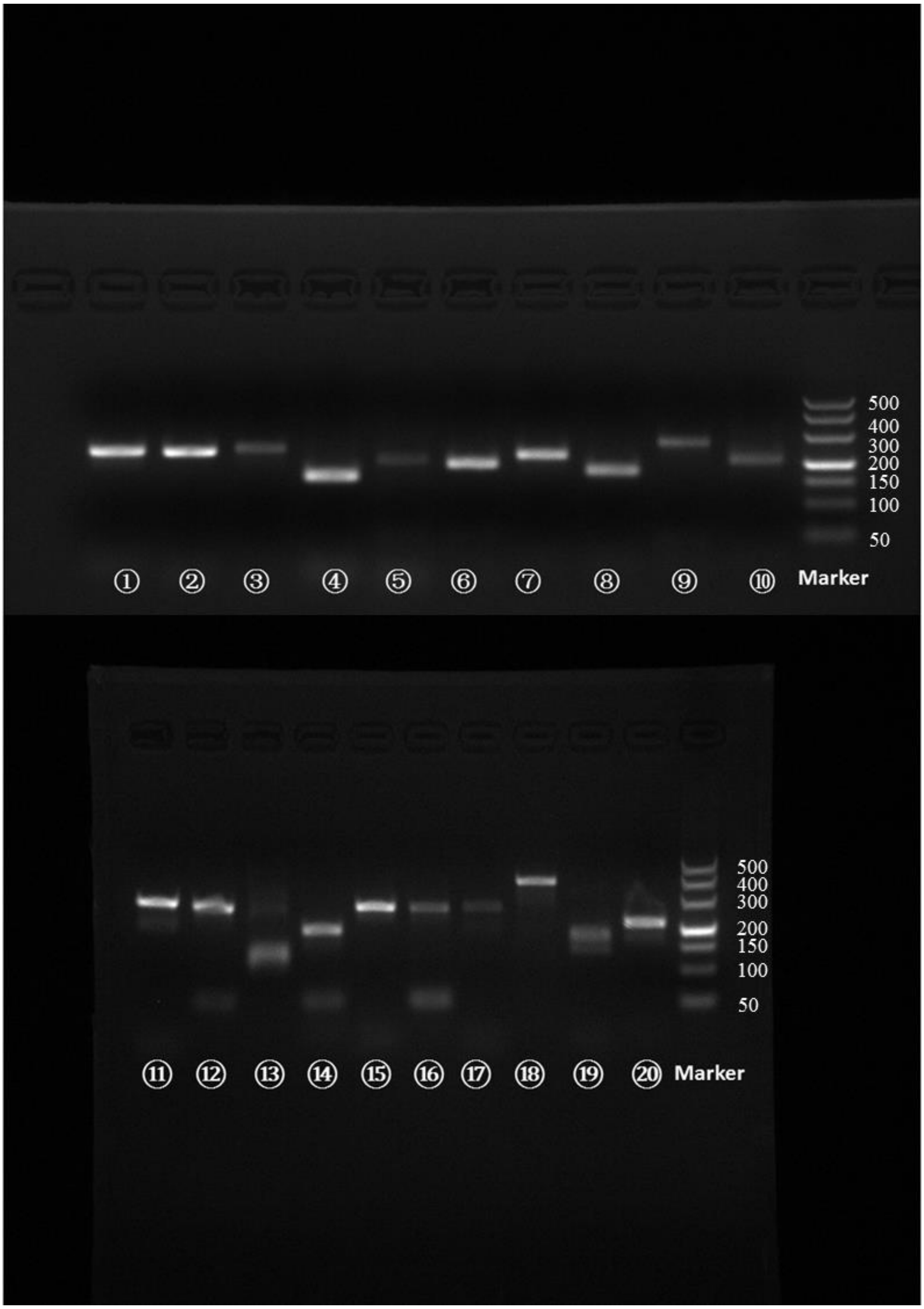

Figure S8. Testis-derived cell-free seminal circRNAs (cfs-circRNAs) (full-length gels and blots of Fig. 4a, 4b).

① SMARCA5-hsa_circ_0001445； ② CREBBP-hsa_circ_0007637；

③ WDR26-hsa_circ_0002322； ④ TBL1XR1-hsa_circ_0068062；

⑤ SAE1-hsa_circ_0007022； ⑥ ZFY-hsa_circ_0001953；

⑦ CPSF6-hsa_circ_0000417； ⑧ STAU2-hsa_circ_0001811；

⑨ ADD3-hsa_circ_0003357； ⑩ ADD3-hsa_circ_0003357;

⑪ LRWD1-hsa_circ_0003327; ⑫ STK31-hsa_circ_0133980;

⑬ circHIST1H2BA-chr6:25727079-25727268; ⑭ circRNF17-chr13:25341410-25356082

⑮ circBRDT-chr1:92446446-92446735; ⑯ circBRDT-chr1:92428275-92433817

⑰ circSTK31-chr13: 23792336-23794093; ⑱ circSTK31-chr13: 23751718-23776697;

⑲ circSMC1B-chr22:45749858-45750995; ⑳ circSPATA16-chr3:172694758-172766884.

.

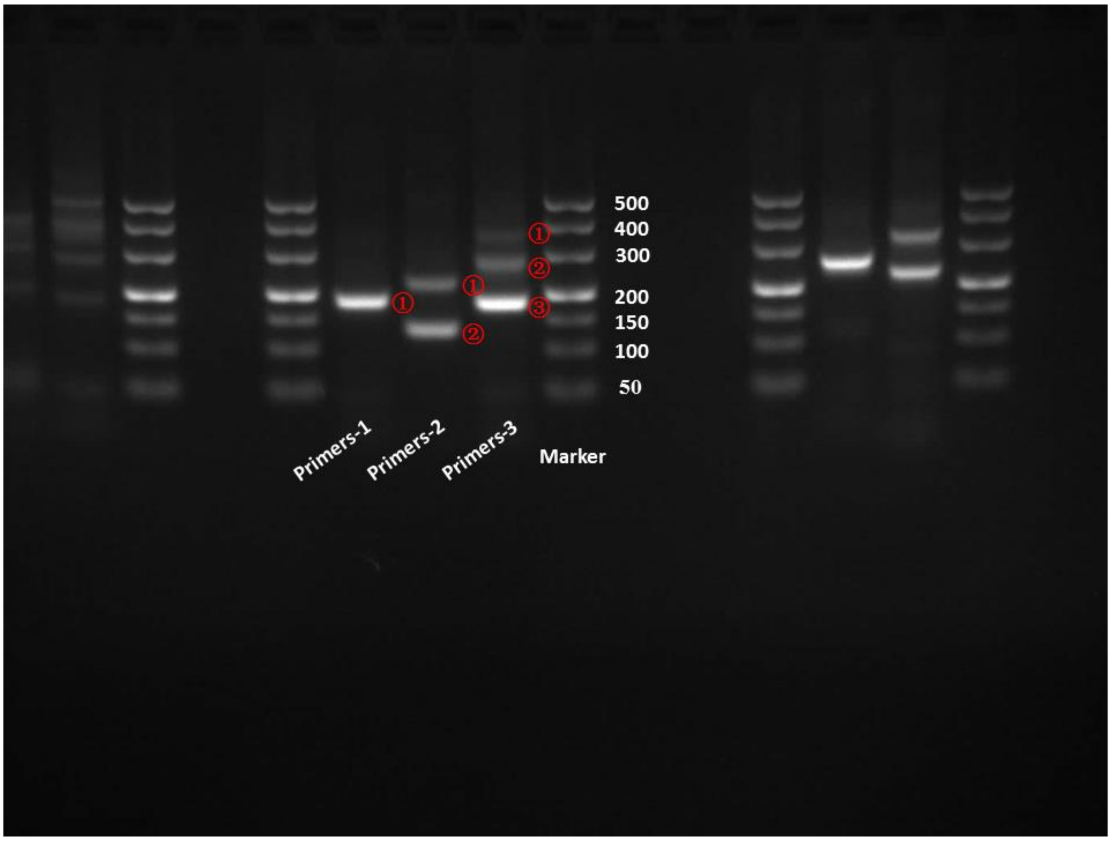

Figure S9. Electrophoretogram for three kinds of circRNAs derived from RNF17 using different primers in RT-PCR (full-length gels and blots of Fig. 3d)

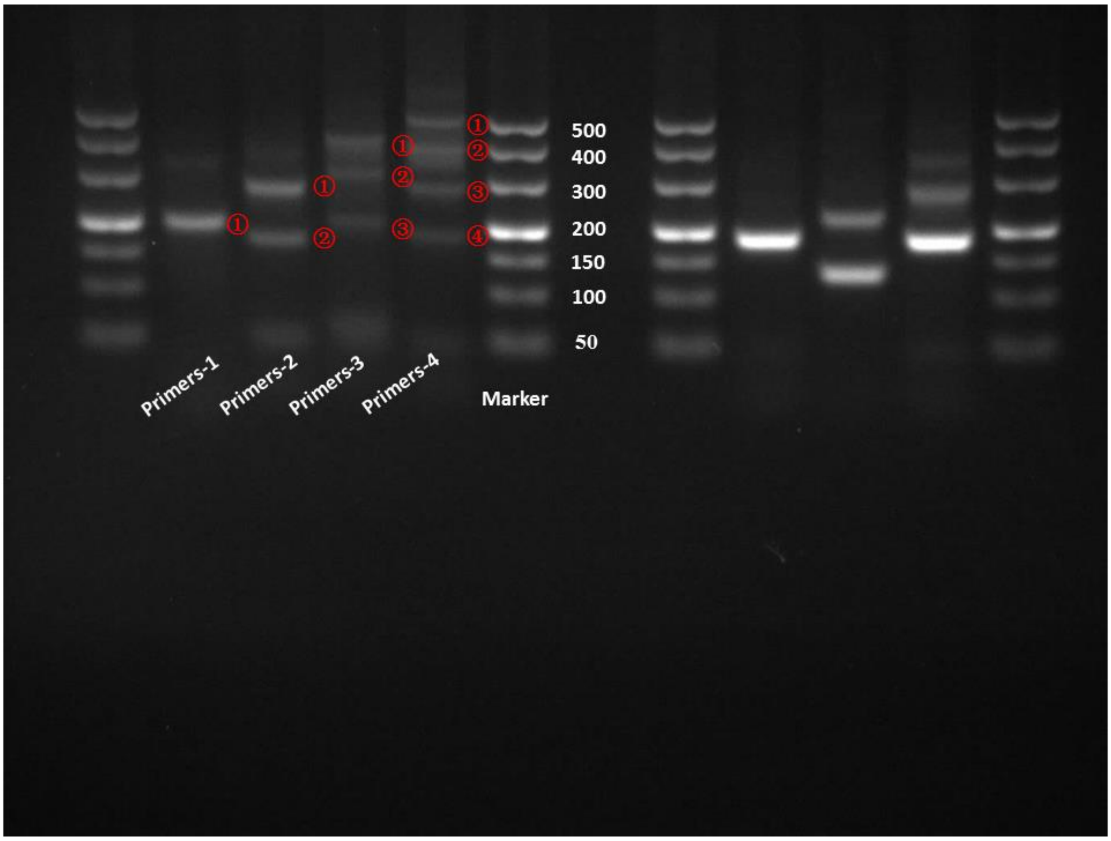

Figure S10. Alternative splicing of SAE1 formed four kinds of circRNAs (full-length gels and blots of Fig. S6b)
